# Management of treatment-resistant acquired perforating dermatosis with apremilast: A novel therapeutic approach

**DOI:** 10.1016/j.jdcr.2025.02.043

**Published:** 2025-03-28

**Authors:** Humza Zaidi, Aziz Khan, Shivani Sinha, Brett Sloan, Donna Aiudi, Gillian Weston

**Affiliations:** a School of Medicine, University of Connecticut, Farmington, Connecticut; b Department of Dermatology, University of Connecticut Health Center, Farmington, Connecticut

**Keywords:** acquired perforating dermatosis (APD), apremilast, chronic kidney disease, diabetes mellitus, inflammatory skin diseases, perforating dermatoses (PD), phosphodiesterase 4 (PDE4) inhibitor, pruritus, treatment-resistant dermatologic conditions

## Introduction

Acquired perforating dermatosis (APD) is a rare skin disorder commonly associated with chronic kidney disease and diabetes mellitus.^1^^,^^2^ The pathogenesis of APD is not completely understood, but it is hypothesized to involve the degeneration and transepidermal elimination of collagen fibers in response to superficial trauma.^3^^,^^4^ APD presents significant management challenges, with treatment strategies primarily focused on addressing the underlying medical conditions and alleviating symptoms.^2^ We present a case of APD resistant to multiple conventional therapies that responded to apremilast, a small molecule phosphodiesterase 4 inhibitor with broad anti-inflammatory properties. This case highlights the potential utility of apremilast in the treatment-resistant cases of APD.

## Case

**Fig 1:**
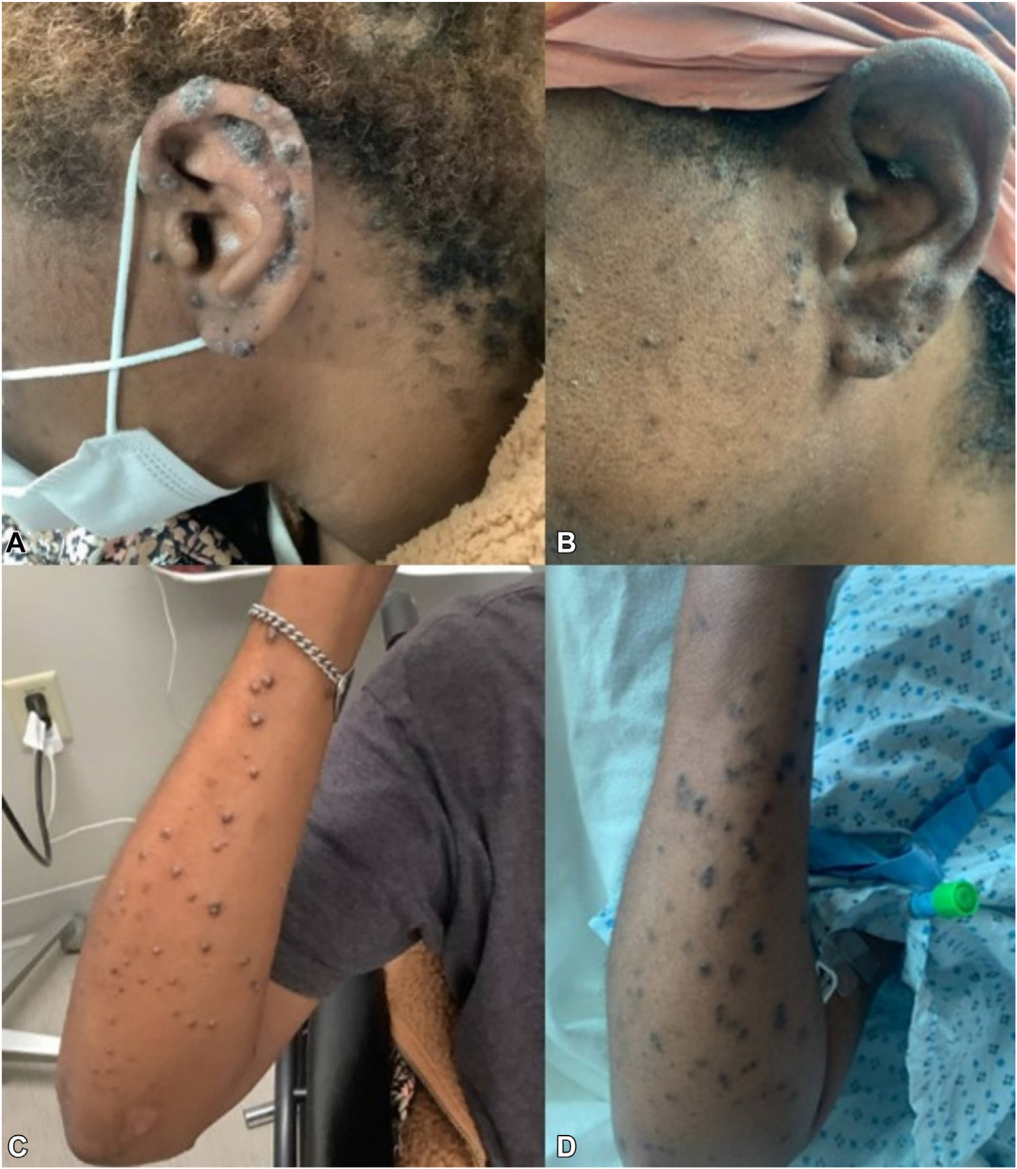
Multiple *dark brown*, hyperkeratotic papules measuring 3-6 mm on the left ear (**A**) and right forearm (**C**) at initial presentation. Significant improvement is observed after 2 months of apremilast treatment on the left ear (**B**) and right forearm (**D**).

**Fig 2:**
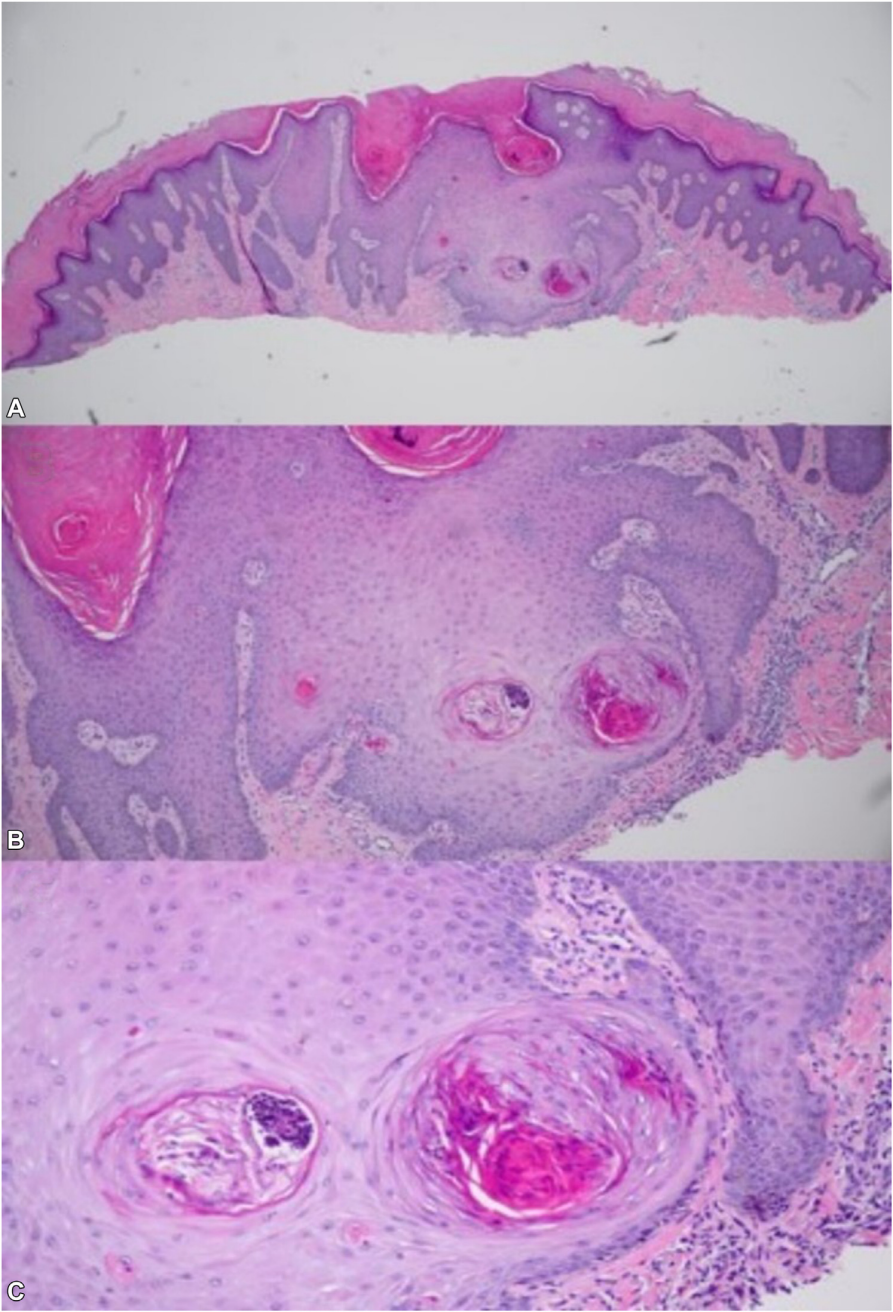
Right knee lesion biopsy with hematoxylin and eosin stain at 4× (**A**), 10× (**B**), and 20× (**C**) magnification levels, showing cup-shaped invagination of the epidermis filled with a plug consisting of keratin and cellular debris, channels with hyperkeratotic plugs and dyskeratotic keratinocytes, as well as neutrophils and degenerated collagen bundles.

A 38-year-old female with a history of diabetes mellitus and end-stage renal disease presented with generalized painful and pruritic lesions of 6 months duration. Initially, the lesions were localized to the left knee, but subsequently spread to involve the entire body, significantly impacting her quality of life. Examination revealed multiple, dark-brown, 3-6 mm hyperkeratotic papules involving the face, ears, back, and the upper and lower extremities (Fig 1, *A* and *C*). A biopsy from a lesion on the right knee with hematoxylin and eosin stain revealed epidermal hyperplasia with endophytic channels containing hyperkeratotic plugs, dyskeratotic keratinocytes, neutrophils, and degenerated collagen bundles (Fig 2, *A*-*C*). Periodic Acid-Schiff, Gomori Methenamine Silver, Acid-Fast Bacillus, and Gram stains were negative for infectious organisms. These histopathological features were consistent with perforating dermatosis. Over a year, various therapeutic options were trialed but failed to control her symptoms. Initial treatment included intralesional triamcinolone, oral antihistamines, triamcinolone 0.1% ointment, clobetasol 0.05% ointment, and tretinoin 0.025% gel without any relief. She also did not respond to therapeutic trials with dupilumab 300 mg subcutaneously every other week for 5 months, narrowband-ultraviolet B phototherapy twice weekly for 4 months, and isotretinoin 20 mg daily for 3 months. She was then initiated on oral apremilast (10 mg daily, titrated up to 30 mg daily) with significant diminution of pruritus, flattening of previously active lesions and reduction in the development of new lesions within 2 months (Fig 1, *B* and *D*). After 1 year of treatment, the patient has continued to remain stable with minimal side effects.

## Discussion

The management of APD presents a significant therapeutic challenge due to its chronic and often treatment-resistant nature.^5^ Traditional treatment strategies involve addressing the underlying medical conditions, commonly chronic kidney disease and diabetes mellitus, and alleviating symptoms. Considering its established efficacy in other inflammatory skin conditions, such as psoriasis and Behcet’s disease,^6^^,^^7^ the patient was started on apremilast.

Apremilast is an inhibitor of phosphodiesterase 4, an enzyme that plays a crucial role in controlling inflammatory responses by regulating cyclic adenosine monophosphate levels. In doing so, apremilast results in the downregulation of proinflammatory cytokines such as tumor necrosis factor-α, interleukins (IL-2, IL-8, IL-12, and IL-23) and interferon-gamma, while promoting the production of anti-inflammatory cytokines such as IL-10.^8^ Phosphodiesterase 4 is widely expressed among various cells, including natural killer cells, macrophage, lymphocytes, and keratinocytes. In addition to modulating cytokine production, apremilast reduces the production of leukotriene B4, nitric oxide synthase, and matrix metalloproteinases, which are involved in processes such as epidermal thickening and neutrophil chemotaxis.^9^ In the context of APD, apremilast’s anti-inflammatory and immunomodulatory effects may help to mitigate local inflammation and reduce the formation of abnormal tissue structures.^3^ Apremilast has a relatively favorable adverse effect profile (including nausea, diarrhea, headaches, weight loss, and mood changes) and requires minimal routine laboratory monitoring.^9^

Our patient experienced remarkable improvement with apremilast, and her APD has remained stable over the past year while continuing the therapy. This outcome aligns with a previous case report, where a patient with Down syndrome and 2 different types of perforating dermatoses --elastosis perforans serpiginosa and perforating folliculitis-- experienced marked improvement on apremilast 30 mg twice daily, with no recurrence after 3 months.^10^ However, unlike the reported case involving elastosis perforans serpiginosa and perforating folliculitis, our patient presented with APD. This outcome highlights the potential of this therapy in refractory cases of APD and warrants further exploration.

## Conflicts of interest

None disclosed.
